# First Total Synthesis of (β-5)-(β-*O*-4) Dihydroxytrimer and Dihydrotrimer of Coniferyl Alcohol (G): Advanced Lignin Model Compounds

**DOI:** 10.3389/fchem.2019.00842

**Published:** 2019-12-09

**Authors:** Amandine L. Flourat, Aurélien A. M. Peru, Arnaud Haudrechy, Jean-Hugues Renault, Florent Allais

**Affiliations:** ^1^URD Agro-Biotechnologies Industrielles (ABI), CEBB, AgroParisTech, Pomacle, France; ^2^Université de Reims Champagne Ardenne, CNRS, Institut de Chimie Moléculaire de Reims, UMR 7312, SFR Condorcet FR CNRS 3417, Reims, France

**Keywords:** lignin models, coniferyl alcohol, monolignol, trimers, total synthesis, C-C coupling, biocatalysis, laccase

## Abstract

To investigate lignin degradation, scientists commonly use model compounds. Unfortunately, these models are most of the time simple β-*O*-4 dimers and do not sufficiently mimic the wide complexity of lignin structure (i.e., aliphatic side chains and robust C-C bonds). Herein, we present a methodology to access advanced lignin models through the first synthesis of two trimers of monolignol **G**—possessing side-chains and both robust β-5 bond and labile β-*O*-4 bond—via a chemo-enzymatic pathway. Key steps were (1) the C-C coupling via laccase-mediated oxidation, (2) the C-O coupling via a simple S_N2_ between a phenolate and a bromoketoester, and (3) a modified Upjohn dihydroxylation or a palladium-catalyzed hydrogenation. (β-5)-(β-*O*-4) dihydroxytrimer and dihydrotrimer of coniferyl alcohol (**G**) were obtained in good global yield, 9 and 20%, respectively, over nine steps starting from ferulic acid.

## Introduction

**Scheme 1:**
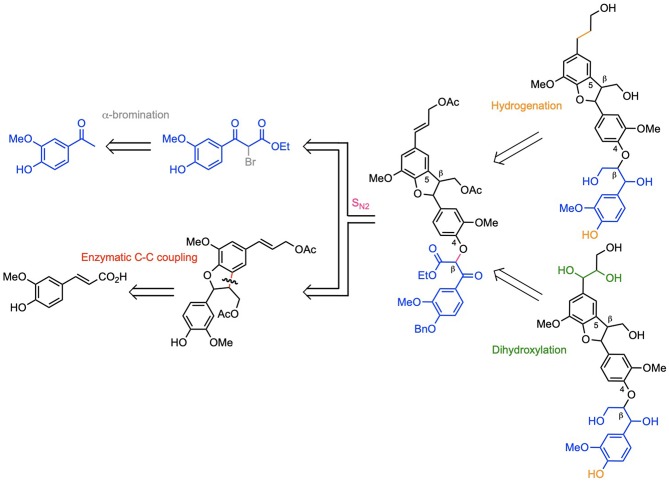
Retrosynthetic routes towards dihydroxy-(β-5)-(β-O-4) trimer and naturally occurring dihydro-(β-5)-(β-O-4) trimer of coniferyl alcohol G.

Lignins are the second most abundant biopolymer on Earth and the first potential source of aromatic compounds. However, due to their tridimensional architecture and the large diversity of linkages between their three principal constitutive units, they are under exploited. In order to provide a better understanding of this extremely complex biopolymer and design efficient degradation processes, models of lignin have been used for a long time. To the best of our knowledge, the first reported synthesis of lignin model compounds emerged in 1952 (Sen, 1952). Recent studies still involved lignin model compounds (Gao et al., 2018; Lee and Yang, 2018; Rinesch and Bolm, 2018; Shimizu et al., 2018). They mainly used (β-*O*-4) dimers without side chains as models to study oxidative cleavage of the ether bond, known as the non-condensed linkage in lignin. Not only such approach does not address the steric effect and electronical interaction due to the side chains, but it also bypasses the robust C-C bond in the lignins, the ones that are responsible for lignin recalcitrance to degradation. Even if (β-*O*-4) linkage is abundant, it seems to us important also to look at the impact of oxidative treatments on condensed bond, such as (β-5) C-C bond, in order to efficiently depolymerize lignins. Recently, Forsythe et al. have described an interesting route to obtain controlled oligomers containing (5-5), (β-5), and (β-*O*-4) linkages but always without functionalization of the side chain (Forsythe et al., 2013). In previous works, we have synthesized model compounds with elaborated side chains to (1) conduct electrochemical study (Cottyn et al., 2015), (2) develop analytical method and immunohistochemistry approaches (Mouterde et al., 2013), or (3) unveil the chemical reactivity of lignosulfonates (Broussard et al., 2016). Such models can be used for numerous applications such as determining energetic level for electronic excitation (e.g., photochemistry), determining preferential pathways of (de)lignification, or estimating the required energy to break specific C-C bonding and release monomers from lignin. Herein, we describe the first total chemo-enzymatic synthesis of both the (β-5)-(β-*O*-4) dihydroxytrimer of monolignol **G** and the naturally occurring dihydrotrimer (Ono et al., 1998; Ye et al., 2016; Jiang et al., 2017). Monolignol **G** is the most widespread in nature, as it is the main constituent of softwoods lignin (>90%) and is present in large amount in hardwood (30–40%) and grasses (35–50%). (Bouxin, 2011) These two trimers will allow comparison of reactivity with (β-5) and (β-*O*-4) dimers and determining the impact of side chain complexity in depolymerization for instance. The chosen synthetic methodology is a convergent pathway starting from ferulic acid and acetovanillone, involving (1) the enzymatic dimerization of coniferyl alcohol (**G**) to obtain the (β-5) C-C bond, (2) the formation of the (β-*O-*4) C-O linkage using nucleophilic substitution (S_N2_) between a phenolate and a bromoketoester, and (3) the dihydroxylation of the double bond to finalize the formation of (β-5)/(β-*O*-4) dihydroxytrimer of monolignol **G**, or the hydrogenation of this double bond to achieve the (β-5)/(β-*O*-4) dihydrotrimer of monolignol **G** (Scheme 1).

## Results and Discussion

**Scheme 2:**
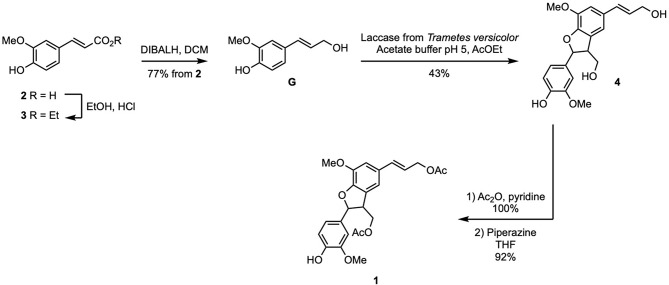
Synthesis of **1** from ferulic acid (**2**).

The synthesis of the two targets started with the preparation of di-acetylated (β-5)-dimer **1** from ferulic acid (**2**), in five steps. Using the strategy previously reported for sinapyl alcohol (Jaufurally et al., 2016), **2** was transformed into ethyl ferulate (**3**) through a Fischer esterification. Subsequent DibalH-mediated reduction of **3** provided coniferyl alcohol (aka monolignol **G**) in 77% overall yield from **2**. Enzymatic dimerization of **G** in the presence of laccase isolated from *Trametes versicolor* (EC.420-150-4) was performed in an emulsion of ethyl acetate and aqueous acetate buffer pH = 5 at room temperature. It is noteworthy to mention that a slow addition of laccase solution via a syringe pump allowed controlling the ratio between **G** and radical species in the reaction medium, thus limiting the formation of oligomers. Only *trans* diastereoisomer of β-5 dimer of **G** (**4**) was obtained as determined by NMR and according to Lou et al. (2018) (43% yield) (data shown in Supplementary Material). The two aliphatic alcohols and the phenol moiety were subsequently acetylated (Ac_2_O, pyridine), prior to specific deacetylation of the latter in the presence of piperazine in THF to provide compound **1** (92% yield, 30% overall yield in five steps starting from ferulic acid) (Scheme 2).

**Scheme 3:**
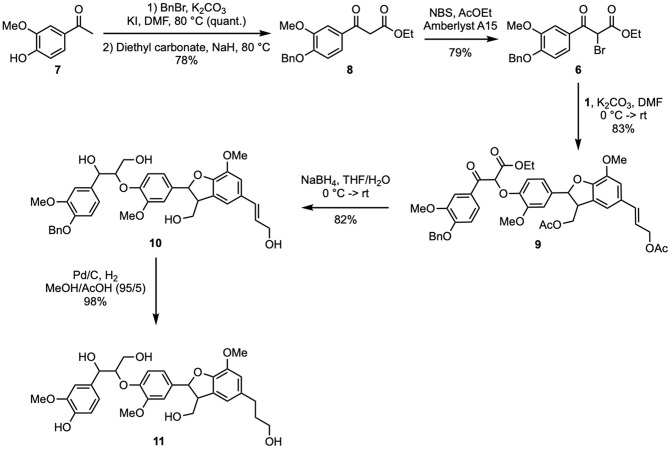
Synthesis of naturally occurring dihydrotrimer (β-5)-(β-O-4) (**11**).

In another hand, the synthesis of bromoketoester of guaiacol (**6**) was performed from acetovanillone (**7**) in three steps (Scheme 3). Benzylation of **7** (BnBr, KI, K_2_CO_3_, DMF, quant.) gave *O*-benzylacetovanillone that was then reacted with diethylcarbonate and sodium hydride to provide the corresponding β-ketoester (**8**) in 78% yield. The latter then underwent bromination (NBS, Amberlyst A15) giving **6** (79% yield, 62% overall yield from **7**).

Formation of the β-*O*-4 bond was achieved through the coupling between **6** and **1** via a S_N2_ process as previously reported by Forsythe et al. (2013) and Kishimoto et al. (2006). It is worth mentioning that, to achieve (β-*O-*4) bond, such S_N2_ was preferred to the aldolization strategy we previously applied for the preparation of (5-5′)-(8′-*O*-4″) dihydrotrimer of ferulic acid (Mouterde et al., 2013), due to easier operation conditions and higher reported yields [49% in acetone (Lancefield and Westwood, 2015) and 97% in DMF (Kishimoto et al., 2006)]. Performing this reaction with potassium carbonate in DMF provided the per-protected (β-5)-(β-*O*-4) trimer of **G** (**9**) in 83% yield (Scheme 3). To access the desired targets, it was necessary to reduce the ketone and ester moieties. To do so, the procedure reported by Patil and Yan (2016) was applied (NaBH_4_, THF/H_2_O) and provided the intermediate **10** in 82% yield. Even if the molecular ion of these adducts cannot be identified in HRMS, probably because of a high reactivity leading to decomposition of the molecule, the disappearance of all peaks between 2.5 and 1.5 ppm and those at 4.24 and 1.21 ppm in the ^1^H NMR spectrum proved the cleavage of the acetates and esters, respectively (data shown in Supplementary Material). Moreover, ^13^C NMR spectrum further confirmed this result and proved also the reduction of the ketone as the peak at 190.0 ppm disappeared.

To access the dihydrotrimer **11**, intermediate **10** was submitted to a palladium-catalyzed hydrogenation step in order to simultaneously cleave the benzyl protecting group and reduce the double bond. It was observed that performing the reaction in ethanol, resulted only in the reduction of the double bond, leaving the benzyl protective group untouched. The cleavage of recalcitrant benzyl moieties being facilitated in highly polar solvent and in the presence of an acid, the successful concomitant debenzylation and reduction of **10** was achieved by using a 95/5 mixture of methanol and acetic acid, providing dihydrotrimer (β-5)-(β-*O*-4) (**11**) in 98% yield (Scheme 3, 67% overall yield from **1**, and 20% from ferulic acid in nine steps). It is worth mentioning that despite many attempt, no HRMS data could be obtained for **11**. Nevertheless, ^1^H & ^13^C NMR analyses proved the reduction of the double bond and the cleavage of the benzyl protecting group.

**Scheme 4:**
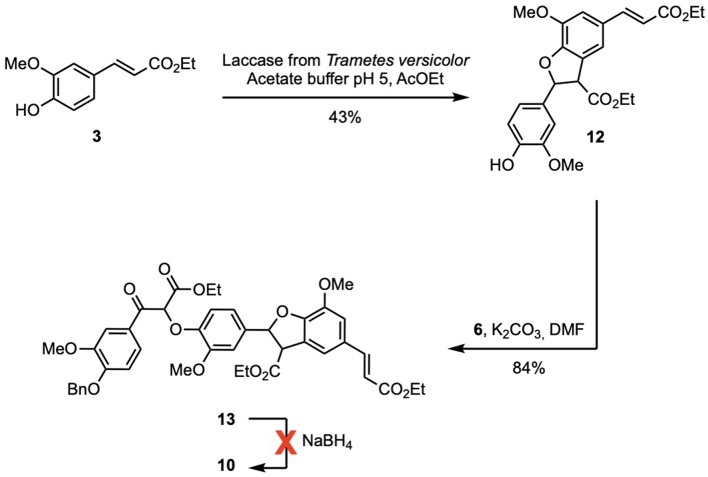
Unsuccessful alternative pathway to **10**.

Another pathway to intermediate **10** was designed to reduce the number of steps. Ethyl ferulate (**3**) was enzymatically dimerized using the same procedure applied for **4** leading to a phenolic diester (**12**) in similar yield. This procedure circumvented the reduction of **3** into **G**, the acetylation and the specific deacetylation. As the phenol moiety was free, the phenolate was directly generated by action of potassium carbonate and reacted with bromoketoester **6** to provide the desired ketotriester (**13**) in excellent yield (84%). The aluminum-mediated reduction (Dibal-H) of **13** proved troublesome due to extreme difficulty to recover the desired product from the quenched reaction mixture. Unfortunately, although there have been reports on the successful NaBH_4_-mediated reduction of aromatic/conjugated esters (Bianco et al., 1988; Boechat et al., 2004; da Costa et al., 2006) the NaBH_4_-mediated reduction of **13** proved unsuccessful even with longer reaction time (4 days) or addition of more NaBH_4_ (up to 20 equivalents) (Scheme 4).

**Scheme 5:**
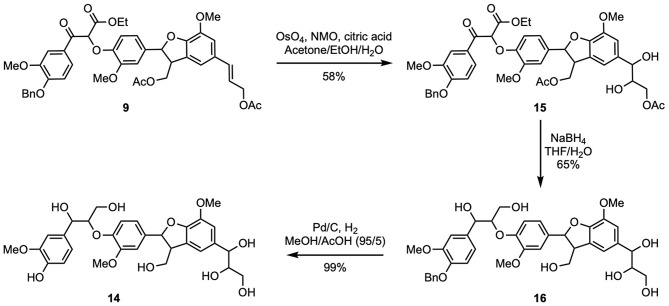
Synthesis of naturally occurring (β-5)-(β-O-4) dihydroxytrimer (**14**).

Finally, in order to access naturally occurring (β-5)-(β-*O*-4) dihydroxytrimer **14**, the double bond in **9** had to be converted into the corresponding 1,2-diol through a dihydroxylation step (Scheme 5). The use of a modified osmium tetroxide-based Upjohn approach (K_2_OsO_4_, NMO, EtOH/H_2_O, citric acid) (Moreaux et al., 2019) allowed the formation of compound **15** (58% yield). The latter was then deprotected in the presence of sodium borohydride to provide **16** in 65% yield. Compound **16** exhibiting a high polarity, its purification through flash chromatography had to be performed in reverse phase. Finally, dihydroxytrimer (β-5)-(β-*O*-4) **14** was obtained after the removal of the benzyl group using the same hydrogenation procedure as previously described. Efficient removal of acetic acid and ethanol was achieved through azeotropic distillation, providing pure **14** in excellent yield (99% overall yield starting from ferulic acid in nine steps).

## Materials and Methods

### Material

Ferulic acid, 1 M diisobutyl aluminum hydride in dichloromethane, benzyl bromide, laccase from *Trametes versicolor* (776 U/g), piperazine, and sodium hydride were purchased from Sigma-Aldrich and used as received. Acetovanillone, sodium borohydride, pyridine, and diethylcarbonate were purchased from TCI and used as received. Palladium on carbon and anhydrous magnesium sulfate were purchased from Acros Organics and used as received. Deuterated solvents were purchased from Euriso-top. Other reagents, salts, and solvents were purchased from VWR.

DMF was dried using mBraun SPS 800. Evaporations were conducted under reduced pressure (Vario Vacuubrand pump) on Buchi R300. Flash chromatographies were performed on a Puriflash 4100 (Interchim) equipped with and pre-packed INTERCHIM PF-30SI-HP (30 μm silica gel) columns. IR analyses were performed on Cary 630 FTIR (Agilent). NMR analyses were recorded on a Bruker Fourier 300. ^1^H NMR spectra of samples were measured on a 300 MHz apparatus, chemicals shifts were reported in parts per million relative to solvent residual peak (CDCl_3_ δ = 7.26 ppm; DMSO-d_6_ δ = 2.50 ppm). ^13^C NMR spectra of samples were recorded at 75 MHz and calibrated on solvent peak (CDCl_3_ δ = 77.16 ppm; DMSO-d_6_ δ = 39.52 ppm).

### Methods

#### Synthesis of Dimer (β-5) (4)

Coniferyl alcohol **G** (3.0 g, 16.6 mmol) was dissolved in ethyl acetate (170 mL) at room temperature, then citrate/phosphate buffer 4.5 (170 mL) was added and the mixture was vigorously stirred (1,000 rpm). Laccase from *Trametes versicolor* (21 mg, 16.3 U/mmol) was dissolved in pH 4.5 buffer (50 mL) and added at 10 mL.h^−1^ to the mixture. At the end of the addition, layers were separated and aqueous layer was extracted twice with AcOEt (75 mL). Organic layers were then combined, washed with brine, dried over anhydrous MgSO_4_, filtered and concentrated to dryness. Product was purified by flash chromatography over silica gel in 10/90 cyclohexane/AcOEt, providing **4** as an orange foam (1.28 g, 43%).

IR (neat): ν = 3,332 (O-H), 2,926 + 2,869 (C=C), 1,610 + 1,516 + 1,496 + 1,461 (C=C arom) cm^−1^.

^1^H NMR (DMSO-d_6_, 300 MHz) δ = 9.02 (s, 1H, H_23_), 6.93 (m, 3H, H_10_ + H_20_ + H_21_), 6.75 (s, 2H, H_6_ + H_17_), 6.46 (d, 1H, *J* = 15.9 Hz, H_4_), 6.21 (dt, 1H, *J* = 15.9 Hz, *J* = 5.4 Hz, H_3_), 5.45 (d, 1H, *J* = 6.9 Hz, H_15_), 5.02 (t, 1H, *J* = 5.4 Hz, H_14_), 4.78, (t, 1H, *J* = 5.7 Hz, H_1_), 4.08 (td, 2H, *J* = 5.4 Hz, *J* = 1.2 Hz, H_2_), 3.79 (s, 3H, H_22_), 3.74 (s, 3H, H_11_), 3.58–3.74 (m, 2H, H_13_), 3.43 (m, 1H, H_12_) ppm.

^13^C NMR (DMSO-d_6_, 75 MHz) δ = 147.6 + 147.1 (Cq, C_8_ + C_19_), 146.4 + 143.7 (Cq, C_7_ + C_18_), 132.3 (Cq, C_16_), 130.5 (Cq, C_5_), 129.5 (Cq, C_9_), 129.0 (Cs, C_4_), 128.0 (Cs, C_3_), 118.5 + 115.3 (Cs, C_6_ + C_17_), 114.9 (Cs, C_20_), 110.3 (Cs, C_10_ + C_21_), 87.2 (Cs, C_15_), 62.9 (Cd, C_13_), 61.7 (Cd, C_2_), 55.6 (Ct, C_11_ + C_22_), 53.0 (Cs, C_12_) ppm.

HRMS (EI): Calcd for C_20_H_22_O_6_ [M]: 358.1416 and for C_20_H_23_${\text{O}}_{6}^{+}$ [M+H]^+^: 359.1489, found 359.1566.

#### Synthesis of Tri-O-Acetylated Dimer (β-5) (5)

**4** (2.6 g, 7.25 mmol) was dissolved in pyridine (18 mL) and acetic anhydride (11 mL, 109 mmol, 5 equiv/function) was added. The reaction was stirred at room temperature overnight. Reaction medium was poured into acidified iced water. The precipitate was filtered off and rinsed with water until neutral pH. After drying, **5** was recovered in quantitative yield and used in the next step without further purification.

IR (neat): ν = 2,940 (C-C), 1,733 (C = O), 1,603 + 1,506 + 1,462 + 1,421 + 1,366 + 1,332 (C=C arom), 1,214 + 1,187 + 1,146 + 1,127 (C-O) cm^−1^.

^1^H NMR (CDCl_3_, 300 MHz) δ = 6.98 (m, 3H, H_10_ + H_20_ + H_21_) 6.88 (m, 2H, H_6_ + H_17_), 6.59 (m, 1H, H_4_), 6.15 (dt, 1H, *J* = 15.6 Hz, *J* = 6.6 Hz, H_3_), 6.54 (d, 1H, *J* = 6.6 Hz, H_15_), 4.70 (dd, 2H, *J* = 6.6 Hz, 1.2 Hz, H_2_), 4.26–4.47 (m, 2H, H_13_), 3.91 (s, 3H, H_22_), 3.78 (m, 4H, H_15_ + H_11_), 2.30 (s, 3H, H_26_), 2.09 (s, 3H, H_24_), 2.05 (s, 3H, H_25_) ppm.

^13^C NMR (CDCl_3_-d_6_) δ = 171.0 + 170.8 (Cq, C_14_ + C_1_), 169.0 (Cq, C_23_), 151.3 (Cq, C_18_), 148.2 (Cq, C_8_), 144.4 (Cq, C_7_), 139.7 + 139.4 (Cq, C_16_ + C_19_), 134.3 (Cs, C_4_), 130.7 (Cq, C_5_), 127.3 (Cq, C_9_), 122.9 (Cs, C_20_), 121.3 (Cs, C_3_), 118.2 (Cs, C_21_), 115.4 (Cs, C_10_), 110.6 (Cs, C_17_), 109.9 (Cs, C_6_), 88.1 (Cs, C_15_), 65.4 (Cd, C_13_), 65.2 (Cd, C_2_), 56.0 + 55.9 (Ct, C_11_ + C_22_), 50.5 (Cs, C_12_), 20.6–21.1 (Ct, C_24_ + C_25_ + C_26_) ppm.

HRMS (EI): Calcd for C_26_H_28_O_9_ [M]: 484.1733 and for C_26_H_28_O_9_Na^+^ [M+Na]^+^: 507.1631 found 507.1637.

#### Synthesis of diO-Acetylated Dimer (β-5) (1)

**5** (7.25 mmol) was dissolved in THF (70 mL) and piperazine (1.87 g, 21.75 mmol, 3 equiv) was added. Reaction mixture was stirred 4 h at room temperature. After completion, THF was removed under reduced pressure. The crude mixture was dissolved in AcOEt (50 mL), washed with HCl 1 M (3^*^15 mL) and brine (20 mL), dried over anhydrous MgSO_4_, filtered and concentrated. After purification by flash chromatography in 40/60 cyclohexane/AcOEt, **1** was recovered as colorless oil (2.95 g, 92%).

IR (neat): ν = 3,443 (OH), 2,939 (C-C), 1,732 (C = O), 1,601 + 1,515 + 1,493 + 1,460 + 1,423 + 1,364 + 1,331 (C=C arom), 1,216 + 1,144 (C-O) cm^−1^.

^1^H NMR (CDCl_3_, 300 MHz) δ = 6.88 (m, 5H, H_6_ + H_10_ + H_17_ + H_20_ + H_21_), 6.60 (d, 1H, *J* = 15.9 Hz, H_4_), 6.16 (dt, 1H, *J* = 15.9 Hz, *J* = 6.6 Hz, H_3_), 5.65 (s, 1H, H_23_), 5.47 (d, 1H, *J* = 7.5 Hz, H_15_), 4.71 (dd, *J* = 1.2 Hz, *J* = 6.6 Hz, 2H, H_2_) 4.37 (m, 2H, H_13_), 3.91 (s, 3H, H_22_), 3.86 (s, 3H, H_11_), 3.77 (m, 1H, H_12_), 2.10 (s, 3H, H_24_), 2.03 (s, 3H, H_25_) ppm.

^13^C NMR (CDCl_3_-d_6_, 75 MHz) δ = 171.1 (Cq, C_14_), 171.0 (Cq, C_1_), 148.4 (Cq, C_8_), 146.8 (Cq, C_18_), 146.0 (Cq, C_19_), 144.5 (Cq, C_7_), 134.5 (Cq, C_16_), 132.3 (Cs, C_4_), 130.6 (Cq, C_5_), 127.8 (Cq, C_9_), 121.2 (Cs, C_3_), 119.7 (Cs, C_21_), 115.4 (Cs, C_20_), 114.4 (Cs, C_10_), 110.6 (Cs, C_17_), 108.7 (Cs, C_6_), 89.0 (Cs, C_15_), 65.4 (Cd, C_2_ + C_13_), 56.1 (Ct, C_11_ + C_22_), 50.4 (Cs, C_12_), 21.2 (Ct, C_24_), 21.0 (Ct, C_25_) ppm.

HRMS (EI): Calcd for C_24_H_26_O_8_ [M]: 442.1628 and for C_24_H_27_${\text{O}}_{8}^{+}$ [M+H]^+^: 443.1700 found 443.1867.

#### Synthesis of 9

Under N_2_, **1** (1.4 g, 3.16 mmol) was dissolved in dry DMF (10 mL) and K_2_CO_3_ (0.66 g, 4.75 mmol, 1.5 equiv) was added (reaction turned yellow). After cooling to 0°C, a solution of **6** (1.9 g, 4.75 mmol, 1.5 equiv) in DMF (9 mL) was added dropwise. Reaction mixture was stirred at room temperature for 3 h, then quenched with water (50 mL) and extracted with AcOEt (3^*^50 mL). Organic layers were combined, washed with brine, dried over anhydrous MgSO_4_, filtered and concentrated. After purification by flash chromatography in 5/5 cyclohexane/AcOEt, **9** was recovered as an oil (2.04 g, 83%).

IR (neat): ν = 2,953 (C-C), 1,734 (C = Oester), 1,676 (C = Oketone), 1,593 + 1,508 + 1,459 + 1,420 + 1,379 + 1,332 (C=Carom), 1,209 + 1,143 (C-O) cm^−1^.

^1^H NMR (CDCl_3_, 300 MHz) δ = 7.77 (dd, *J* = 8.4 Hz, *J* = 2.1 Hz, 1H, H_31_), 7.68 (d, *J* = 2.1 Hz, 1H, H_27_), 7.27–7.45 (m, 5H, H_35_ + H_36_ + H_37_), 6.74–6.94 (m, 6H, H_6_ + H_10_ + H_17_ + H_20_ + H_21_ + H_30_), 6.59 (d, *J* = 15.6 Hz, 1H, H_4_), 6.15 (dt, *J* = 15.6 Hz, *J* = 6.6 Hz, 1H, H_3_), 5.73 (s, 1H, H_23_), 5.47 (d, *J* = 6.9 Hz, 1H, H_15_), 5.23 (s, 2H, H_33_), 4.71 (d, *J* = 6.6 Hz, 2H, H_2_), 4.43 (m, 1H, H_13a_), 4.24 (m, 3H, H_13b_ + H_38_), 3.92 (s, 3H, H_32_), 3.90 (s, 3H, H_11_), 3.75 (m, 4H, H_22_ + H_12_), 2.09 (s, 3H, H_41_), 2.01 (s, 3H, H_40_), 1.21 (t, *J* = 7.2 Hz, 3H, H_39_) ppm.

^13^C NMR (CDCl_3_, 75 MHz) δ = 190.0 (Cq, C_25_), 170.9–171.3 (Cq, C_1_ + C_14_), 167.0 (Cq, C_24_), 153.3 (Cs, C_29_), 150.7 (Cs, C_18_), 150.6 (Cs, C_8_), 149.5 (Cs, C_28_), 148.2 (Cs, C_34_), 146.3 (Cs, C_19_), 144.5 (Cs, C_7_), 136.2 (Cs, C_5_ + C_16_), 134.4 (Cs, C_4_), 130.7 (Cs, C_9_), 128.8 (Cs, C_36_), 128.3 (Cs, C_26_), 127.5 (Cs, C_37_), 127.3 (Cs, C_35_), 124.8 (Cs, C_31_), 121.3 (Cs, C_3_), 118.7 (Cs, C_20_), 118.3 (Cs, C_21_), 115.4 (Cs, C_10_), 112.1 (Cs, C_30_), 112.0 (Cs, C_27_), 110.5 (Cs, C_17_) 110.4 (Cs, C_6_), 88.4 (Cs, C_15_), 82.7 (Cs, C_23_), 70.9 (Cd, C_33_), 65.3 (Cd, C_2_ + C_13_), 62.3 (Cd, C_38_), 56.0–56.1 (Ct, C_11_ + C_22_ + C_32_), 50.4 (Cs, C_12_), 21.2 (Ct, C_41_), 20.9 (Ct, C_40_), 14.3 (Ct, C_39_) ppm.

HRMS (EI): Calcd for C_43_H_44_O_13_ [M]: 768.2782 and for C_43_H_44_O_13_Na^+^ [M+Na]^+^: 791.2680 found 791.2668.

#### Synthesis of 10

**9** (700 mg, 0.91 mmol) was dissolved in THF (7.6 mL) and water was added (2.4 mL). At 0°C, NaBH_4_ (344 mg, 9.1 mmol, 10 equiv) was added by portions. At the end of the addition, reaction mixture was stirred for two more hours at room temperature, then quenched by a saturated aqueous solution of NH_4_Cl (10 mL) and extracted with AcOEt (2^*^30 mL). Organic layers were combined, washed with brine, dried over anhydrous MgSO_4_, filtered and concentrated. After purification by flash chromatography in 100% AcOEt, **10** was recovered as an oil (432 mg, 82%).

IR (neat): ν = 3,346 (O-H), 2,934 (C-C), 1,595 + 1,508 + 1,459 + 1,420 + 1,379 + 1,326 (C=Carom), 1,257 + 1,217 + 1,137 (C-O) cm^−1^.

^1^H NMR (DMSO-d_6_, 300 MHz) δ = 7.30–7.44 (m, 5H, H_35_ + H_36_ + H_37_), 6.75–7.15 (m, 8H, H_6_ + H_10_ + H_17_ + H_20_ + H_21_ + H_27_ + H_30_ + H_31_), 6.60 (d, *J* = 15.6 Hz, 1H, H_4_), 6.21 (dt, *J* = 15.6 Hz, *J* = 6.6 Hz, 1H, H_3_), 5.49 (m, 1H, H_15_), 5.45 (m, 1H, H_25_), 5.05 (m, 3H, H_33_ + H_39_), 4.74 (m, 1H, H_23_), 4.64 (m, 2H, H_2_), 4.33 (m, 1H, H_24a_), 3.80 (s, 3H, H_11_), 3.73 (m, 4H, H_32_ + H_1_), 3.67 (s, 3H, H_22_), 3.60 (m, 3H, H_13_ + H_24b_), 3.42 (m, 1H, H_12_) ppm.

^13^C NMR (DMSO, 75 MHz) δ = 150.0 (Cq, C_8_), 148.9 (Cq, C_19_), 148.3 (Cq, C_28_), 148.1 (Cq, C_18_), 147.2 (Cq, C_29_), 144.2 (Cq, C_7_), 137.8 (Cq, C_34_), 135.7 (Cq, C_16_), 134.4 (Cq, C_5_), 134.3 (Cs, C_4_), 130.1 (Cq, C_9_), 129.9 (Cq, C_26_), 128.8 (Cs, C_36_ + C_37_), 128.2 (Cs, C_35_), 121.4 (Cs, C_3_), 119.6 (Cs, C_31_), 118.5 (Cs, C_21_), 116.0 (Cs, C_10_), 115.7 (Cs, C_20_), 113.2 (Cs, C_30_), 111.8 (Cs, C_27_), 111.0 (Cs, C_17_) 110.8 (Cs, C_6_), 87.5 (Cs, C_15_), 84.0 (Cs, C_25_), 71.9 (Cs, C_23_), 70.3 (Cd, C_33_), 65.1 (Cd, C_2_), 60.4 (Ct, C_24_), 60.2 (Ct, C_13_), 56.1 (Ct, C_11_ + C_22_), 55.8 (Ct, C_32_), 53.5 (Cs, C_12_) ppm.

#### Synthesis of 11

**10** (250 mg, 0.38 mmol) was dissolved in methanol/acetic acid (95/5) mixture (5 mL). Reaction medium was purged with nitrogen during 15 min, then palladium on charcoal (25 mg, 10% w/w) was added. The reaction was put under dihydrogen flux overnight at room temperature. Upon completion, reaction medium was purged with nitrogen during 20 min, filtered through PTFE syringe filter, rinsed twice with 5 mL of ethanol and concentrated. **11** was recovered as a foam (217 mg, 98%) without the need of further purification.

IR (neat): ν = 3,388 (O-H), 2,934 (C-C), 1,602 + 1,509 + 1,460 + 1,421 + 1,366 (C=Carom), 1,258 + 1,210 + 1,138 (C-O) cm^−1^.

^1^H NMR (DMSO-d_6_, 300 MHz) δ = 8.75 (s, 1H, H_33_), 7.02–6.64 (m, 8H, H_6_ + H_10_ + H_17_ + H_20_ + H_21_ + H_27_ + H_30_ + H_31_), 5.43 (m, 1H, H_15_), 5.28 (m, 1H, H_25_), 5.00 (s, 1H, H_35_), 4.70 (m, 1H, H_23_), 4.59 (s, 1H, H_14_), 4.44 (s, 1H, H_1_), 4.29 (m, 1H, H), 3.77–3.69 (m, 12H, H_11_ +H_22_ +H_32_ +H), 3.59 (m, 4H, H), 3.40 (m, 8H, H_2_ + H_4_), 1.69 (m, 2H, H_3_) ppm.

^13^C NMR (DMSO, 75 MHz) δ = 150.4, (Cq, C_8_), 148.2 (Cq, C_19_), 147.4 (Cq, C_28_), 146.1 (Cq, C_18_), 145.9 (Cq, C_29_), 143.9 (Cq, C_7_), 140.0 (Cq, C_16_), 134.6 (Cq, C_5_) 133.7 (Cq, C_9_), 128.2 (Cq, C_26_), 119.9 (Cs C_21_ + C_31_), 116.9 (Cs, C_10_ + C_20_), 112.9 (Cs, C_30_ + C_27_), 111.0 (Cs, C_17_ + C_6_), 95.5 (Cs, C_15_), 84.0 (Cs, C_25_), 72.0 (Cs, C_23_), 63.8 (Cd, C_2_), 63.5 (Ct, C_24_), 60.4 (Ct, C_13_), 56.1 (Cs, C_11_ + C_22_ + C_32_), 55.9 (Cs, C_12_) 31.8 (Cd, C_3_), 30.6 (Cd, C_4_) ppm.

#### Synthesis of 15

**9** (840 mg, 1.1 mmol) and citric acid (275 mg, 1.3 mmol) were dissolved in a mixture of acetone (3 mL), ethanol (2 mL) and water (1.5 mL). Osmium tetroxide solution 4% in water (170 μL, 27 μmol) was added and then N-methyl morpholine solution 0.5 M in water (270 μL, 1.3 mmol) was added too. The reaction media was stirred overnight, quenched with Na_2_S_2_O_3_ (10 mL) and extracted thrice with ethyl acetate (10 mL). Organic layers were combined washed with water and brine, dried over anhydrous MgSO_4_, filtered and concentrated. Flash purification was conducted on silica gel with cyclohexane/AcOEt (1/1) and then AcOEt (100%) to afford 512 mg of pure **15** as an oil (58%).

IR (neat): ν = 3,456 (O-H), 2,937 (C-C), 1,734 (C = O ester), 1,675 (C = O ketone), 1,592 + 1,508 + 1,454 + 1,419 + 1,368 (C=C arom), 1,209 + 1,135 (C-O) cm^−1^.

^1^H NMR (CDCl_3_, 300 MHz) δ = 7.77 (dd, *J* = 8.4 Hz, *J* = 2.1 Hz, 1H, H_31_), 7.68 (d, *J* = 2.1 Hz, 1H, H_27_), 7.31–7.44 (m, 5H, H_35_ + H_36_ + H_37_), 6.82–6.93 (m, 6H, H_6_ + H_10_ + H_17_ + H_20_ + H_21_ + H_30_), 5.73 (s, 1H, H_23_), 5.47 (d, *J* = 6.9 Hz, 1H, H_15_), 5.23 (s, 2H, H_33_), 4.57 (m, 1H, H), 4.39 (m, 1H, H), 4.21–4.35 (m, 3H, H_13b_ + H_38_), 4.12 (m, 1H, H), 3.87–4.01 (m, 9H, H), 3.78 (m, 4H, H_22_ + H_12_), 2.10 (s, 3H, H_41_), 2.00 (s, 3H, H_40_), 1.21 (t, *J* = 7.2 Hz, 3H, H_39_) ppm.

^13^C NMR (CDCl_3_, 75 MHz) δ = 190.0 (Cq, C_25_), 170.9 and 171.4 (Cq, C_1_+ C_14_), 167.0 (Cq, C_24_), 153.3 (Cs, C_29_), 150.6 (Cs, C_18_), 149.5 (Cs, C_8_), 149.5 (Cs, C_28_), 148.2 (Cs, C), 146.3 (Cs, C_19_), 144.5 (Cs, C_7_), 136.2 (Cs, C_34_ + C_5_ + C_16_), 134.4 (Cs, C_4_), 130.7 (Cs, C_9_), 128.8 (Cs, C_36_), 128.3 (Cs, C_26_), 127.5 (Cs, C_37_), 127.4 (Cs, C_35_), 124.8 (Cs, C_31_), 118.8 (Cs, C_20_), 118.3 (Cs, C_21_), 115.0 (Cs, C_10_), 112.1 (Cs, C_30_), 112.0 (Cs, C_27_), 110.9 (Cs, C_17_) 110.4 (Cs, C_6_), 88.4 (Cs, C_15_), 82.7 (Cs, C_23_), 77.4, 74.6, 74.3 (Cd, C_4_), 70.9 (Cd, C_33_), 65.5+ 65.3 (Cd, C_2_+ C_13_), 62.3 (Cd, C_38_), 56.0–56.2 (Ct, C_11_ + C_22_ + C_32_), 50.6 (Cs, C_12_), 21.0 (Ct, C_41_), 20.9 (Ct, C_40_), 14.2 (Ct, C_39_) ppm.

HRMS (EI): Calcd for C_43_H_46_O_15_ [M]: 802.2837 and for C_43_H_46_O_15_Na^+^ [M+Na]^+^: 825.2726 found 825.2734.

#### Synthesis of 16

**15** (794 mg, 0.99 mmol) was dissolved in a THF/water mixture (8.2 mL/2.6 mL). At 0°C, sodium borohydride (375 mg, 9.9 mmol, 10 equiv) was added by portion. The reaction medium was stirred at room temperature during 18 h. THF was removed by distillation under reduce pressure and 2.5 mL of methanol was added. The crude mixture was purified by flash chromatography over C18-grafted silica gel with water/MeOH (1/1) and then water/ MeOH (1/4). **16** was recovered as a white foam (448 mg, 65%).

IR (neat): ν = 3,344 (O-H), 2,932 + 2,873 (C-C), 1,602 + 1,507 + 1,451 + 1,419 + 1,321 (C=C arom), 1,258 + 1,213 + 1,135 (C-O) cm^−1^.

^1^H NMR (DMSO, 300 MHz) δ = 7.31–7.41 (m, 5H, H_35_ + H_36_ + H_37_), 6.81–7.06 (m, 8H, H_6_ + H_10_ + H_17_ + H_20_ + H_21_ + H_27_ + H_30_ + H_31_), 5.38–5.47 (m, 2H, H_23_ +H_41_), 5.04 (s, 3H, H_33_ + H_1_), 4.99 (t, *J* = 4.5 Hz, 1H, H_39_), 4.76 (m, 1H, H_3_), 4.66 (m, 1H, H_14_), 4.53 (m, 1H, H_38_), 4.45 (m, 2H, H_25_ + H_40_), 4.33 (m, 1H, H_1_), 3.68–3.77 (m, 10H, H_11_ + H_22_ + H_32_ + H_13a_), 3.60 (m, 3H, H_2_ + H_13b_), 3.43–3.48 (m, 2H, H_12_ + H_24a_), 3.31 (m, 1H, H_4_), 3.19 (m, 1H, H_24b_) ppm.

^13^C NMR (DMSO, 75 MHz) δ = 149.6 (Cq, C_7_), 148.5 (Cq, C_18_), 147.8 (Cq, C_8_), 146.8 (Cq, C_28_), 146.3 (Cq, C_29_), 143.0–143.1 (Cq, C_19_), 137.4 (Cq, C_34_), 136.8 (Cq, C_16_), 135.3 (Cq, C_26_), 134.4 (Cs, C_5_), 128.4 (Cs, C_36_ +Cq, C_9_), 127.8 (Cs, C_37_ + C_35_), 119.2 (Cs, C_31_), 118.0 (Cs, C_21_), 115.1–115.4 (Cs, C_10_), 112.9 (Cs, C_30_), 11.4 (Cs, C_17_), 111.0–111.4 (C_20_), 110.4 (C_6_), 86.8 (Cs, C_23_), 83.5 (Cs, C_15_), 75.9 (Cs, C_4_), 72.8–73.0 (Cs, C_25_), 71.5 (Cs, C_3_), 69.9 (Cd, C_33_), 63.2 (Cd, C_13_), 62.7 (cd, C_24_), 60.0 (Cd, C_2_), 55.7 and 55.4 (Ct, C_22_ + C_32_ + C_11_), 53.5 (Cs, C_12_) ppm.

HRMS (EI): Calcd for C_37_H_42_O_12_ [M]: 678.7310 and for C_37_H_42_O_12_Na^+^ [M+Na]^+^: 701.2574 found 701.2565.

#### Synthesis of 14

**16** (325 mg, 0.48 mmol) was dissolved in a MeOH/AcOH (95/5) mixture (4.8 mL). The reaction medium was purged for 15 min under nitrogen flow before palladium on charcoal (32 mg, 10% w/w) was added. The mixture was then stirred under hydrogen flow overnight. Upon completion, reaction medium was purged with nitrogen during 20 min, filtered through PTFE syringe filter, rinsed twice with 5 mL of ethanol and concentrated. Crude product was submitted to azeotropic evaporation with toluene thrice, then thrice with water to eliminate acetic acid residues. Light yellow oil was recovered (99%).

IR (neat): ν = 3,324 (O-H), 2,933 (C-C), 1,601 + 1,507 + 1,451 + 1,420 (C=C), 1,261 + 1,214 + 1,134 (C-O) cm^−1^.

^1^H NMR (DMSO-d_6_, 300 MHz) δ = 6.52–7.01 (m, 13H, H_6_ + H_10_ + H_17_ + H_20_ + H_21_ + H_27_ + H_30_ +H_31_), 5.45 (m, 1H, H_23_), 4.70 (m, 3H, H_3_ + H_37_ + H_36_), 4.39 (m, 3H, H_25_), 4.24 (m, 2H, H_15_), 3.63–3.77 (m, 14H, H_11_ + H_22_ + H_32_ + H_13a_), 3.08–3.55 (m, 14H, H_2_ + H_3_ + H_4_ + H_12_ + H_13b_ +H_23_ + H_24_) ppm.

^13^C NMR (DMSO, 75 MHz) δ = 149.6–145.5 (Cq, C_7_ + C_18_ + C_8_ + C_28_ + C_29_), 142.9–143.1 (Cq, C_19_), 136.8–136.9 (Cq, C_16_), 132.9–134.4 (Cq, C_5_ + C_26_), 128.2–128.4 (Cq, C_9_), 118.0–120.7 (Cs, C_21_ + C_31_), 114.6–115.5 (Cs, C_10_ + C_30_), 113.2–113.3 (Cs, C_27_), 110.4–111.4 (Cs, C_17_ + C_20_), 107.8–108.0 (Cs, C_6_), 86.8 (Cs, C_23_), 83.7 (Cs, C_15_), 75.9–76.1 (Cd, C_4_), 72.8–73.1 (Cs, C_25_), 71.5 (Cs, C_3_), 63.2–64.3 (Cd, C_13_), 62.6 (Cd, C_24_), 59.9–60.0 (Cd, C_2_) 55.4–55.7 (Ct, C_11_ + C_22_ + C_32_), 53.5 (Cs, C_12_) ppm.

HRMS (EI): Calcd for C_30_H_36_O_12_ [M]: 588.2207 and for C_30_H_36_O_12_Na^+^ [M+Na]^+^: 611.2104 found 611.2110.

## Conclusion

The total convergent synthesis of the β-5/β-*O*-4 dihydroxytrimer of monolignol **G** (**11**) and that of the naturally occurring dihydrotrimer (**10**) have been successfully achieved in nine steps and in 9 and 20% yield, respectively, starting from ferulic acid using laccase-mediated dimerization, nucleophilic substitution (S_N2_), and dihydroxylation as key steps.

## Data Availability Statement

All datasets generated for this study are included in the article/Supplementary Material.

## Author Contributions

AF and FA wrote the publication. AP performed main bibliographical research. AF and AP designed and performed experimental work. AH, J-HR, and FA supervised this work and corrected the proof of article.

### Conflict of Interest

The authors declare that the research was conducted in the absence of any commercial or financial relationships that could be construed as a potential conflict of interest.
