# Supplementary material for: First Total Synthesis of (β-5)-(β-O-4) Dihydroxytrimer and Dihydrotrimer of Coniferyl Alcohol (G): Advanced Lignin Model Compounds
Source: Front Chem. 2019 Dec 9;7:842. doi: 10.3389/fchem.2019.00842 (PMC6913187; doi:10.3389/fchem.2019.00842)
Supplement: Supplementary file 1 [file Data_Sheet_1.docx]

Supplementary Material

First total synthesis of (β-5)-(β-*O*-4) trimer and dihydrotrimer of coniferyl alcohol (G): advanced lignin model compounds

Amandine L. Flourat^1,2*^, Aurélien A. M. Peru^1^, Arnaud Haudrechy^2^, Jean-Hugues Renault^2^, Florent Allais^1,*^

^1^ URD Agro-Biotechnologies Industrielles (ABI), CEBB, AgroParisTech, Pomacle, France

^2^ Université de Reims Champagne Ardenne, CNRS, Institut de Chimie Moléculaire de Reims, UMR 7312, SFR Condorcet FR CNRS 3417*,* F-51097 REIMS Cedex, France

*** Correspondence:**Amandine Flourat and Florent Allais
[amandine.flourat@agroparistech.fr](mailto:amandine.flourat@agroparistech.fr) and [florent.allais@agroparistech.fr](mailto:florent.allais@agroparistech.fr)

[1 ^1^H, ^13^C, Cosy NMR and FT-IR spectra Dimer (β-5) (4) 2](#_Toc24971224)

[2 ^1^H, ^13^C NMR and FT-IR spectra tri-*O*-Acetylated dimer (β-5) (5) 6](#_Toc24971225)

[3 ^1^H, ^13^C NMR and FT-IR spectra di-*O*-Acetylated dimer (β-5) (1) 9](#_Toc24971226)

[4 ^1^H, ^13^C, Cosy, HSQC, HMBC NMR and FT-IR spectra Compound 9 12](#_Toc24971227)

[5 ^1^H, ^13^C, Cosy, HSQC, HMBC NMR and FT-IR spectra Compound 10 18](#_Toc24971228)

[6 ^1^H, ^13^C, Cosy, HSQC, HMBC NMR and FT-IR spectra Compound 11 25](#_Toc24971229)

[7 ^1^H, ^13^C, Cosy, HSQC, HMBC NMR and FT-IR spectra Compound 15 31](#_Toc24971230)

[8 ^1^H, ^13^C, Cosy, HSQC, HMBC NMR and FT-IR spectra Compound 16 37](#_Toc24971231)

[9 ^1^H, ^13^C, Cosy, HSQC, HMBC NMR and FT-IR spectra Compound 14 43](#_Toc24971232)

# ^1^H, ^13^C, Cosy NMR and FT-IR spectra Dimer (β-5) (4)

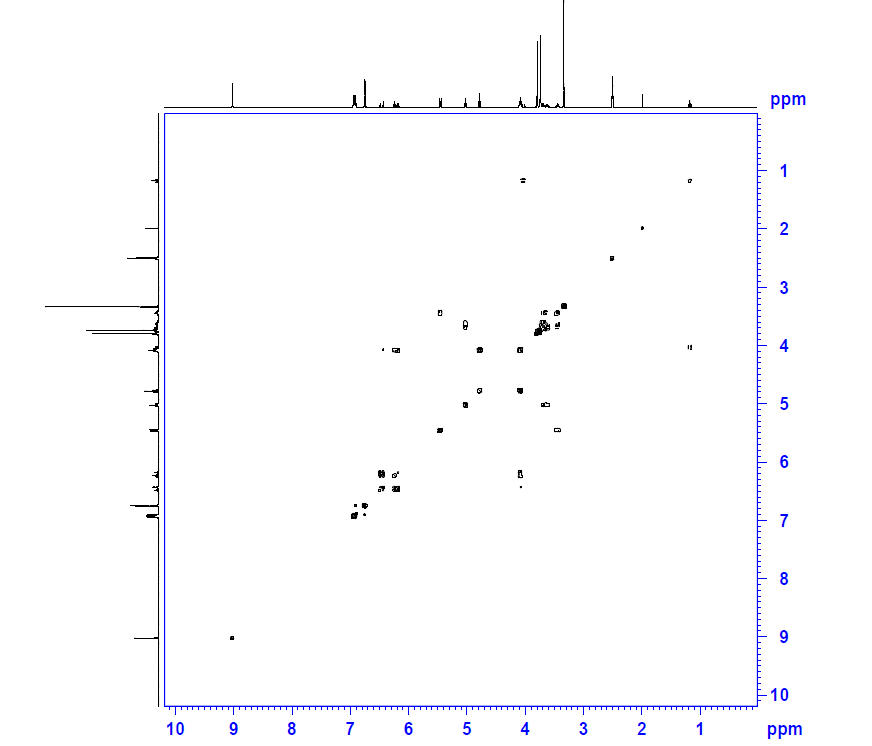


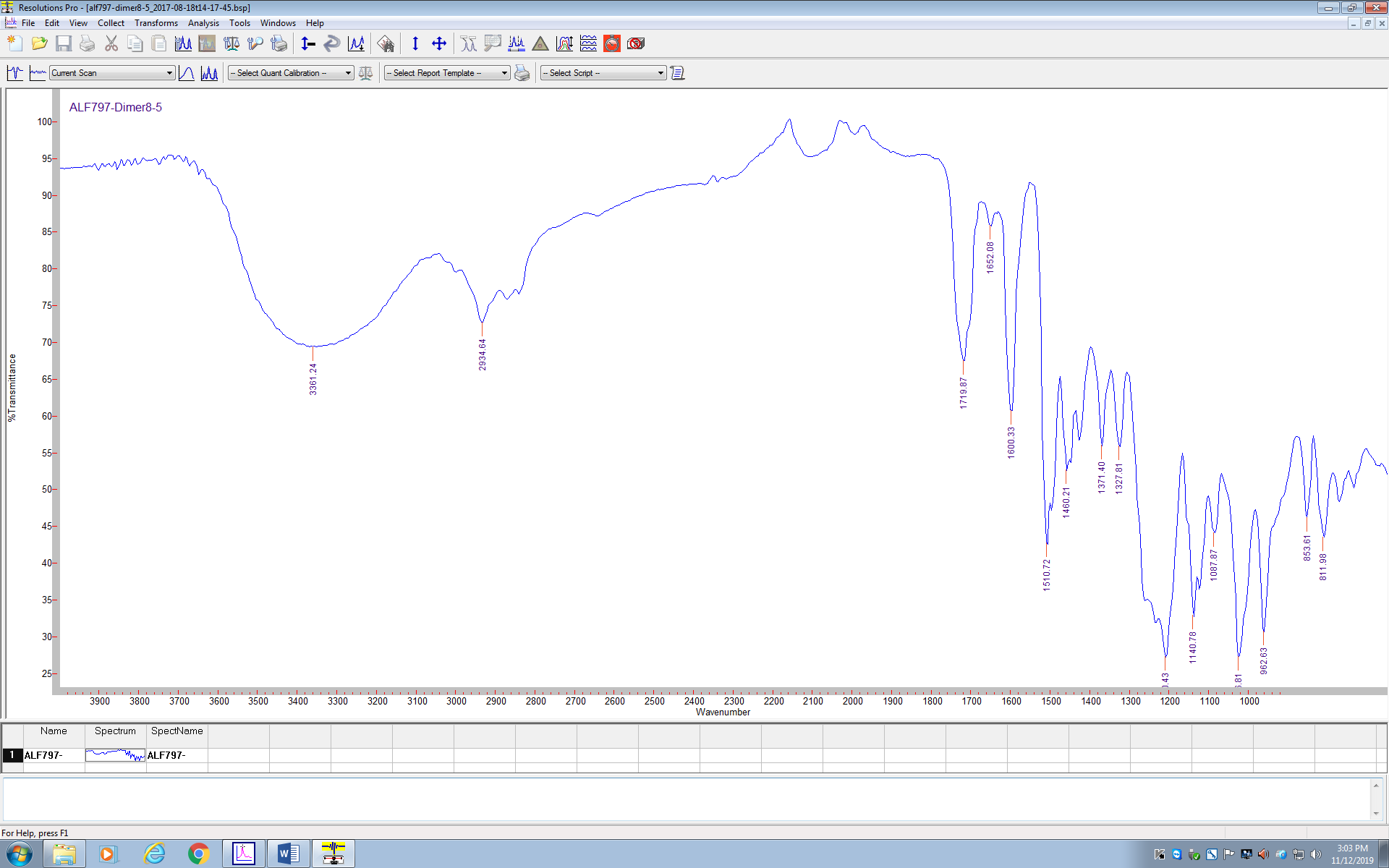


# ^1^H, ^13^C NMR and FT-IR spectra tri-*O*-Acetylated dimer (β-5) (5)

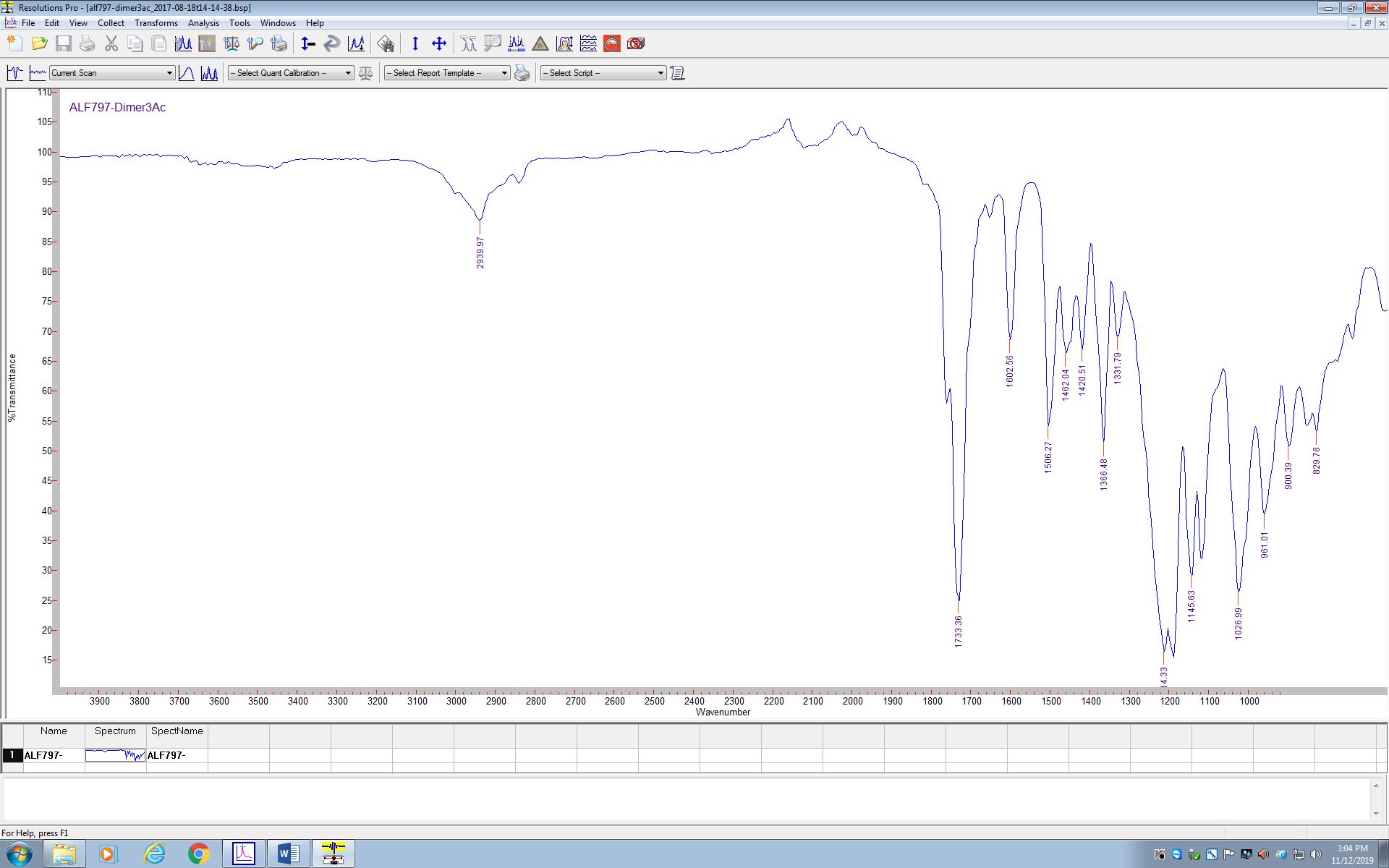


# ^1^H, ^13^C NMR and FT-IR spectra di-*O*-Acetylated dimer (β-5) (1)

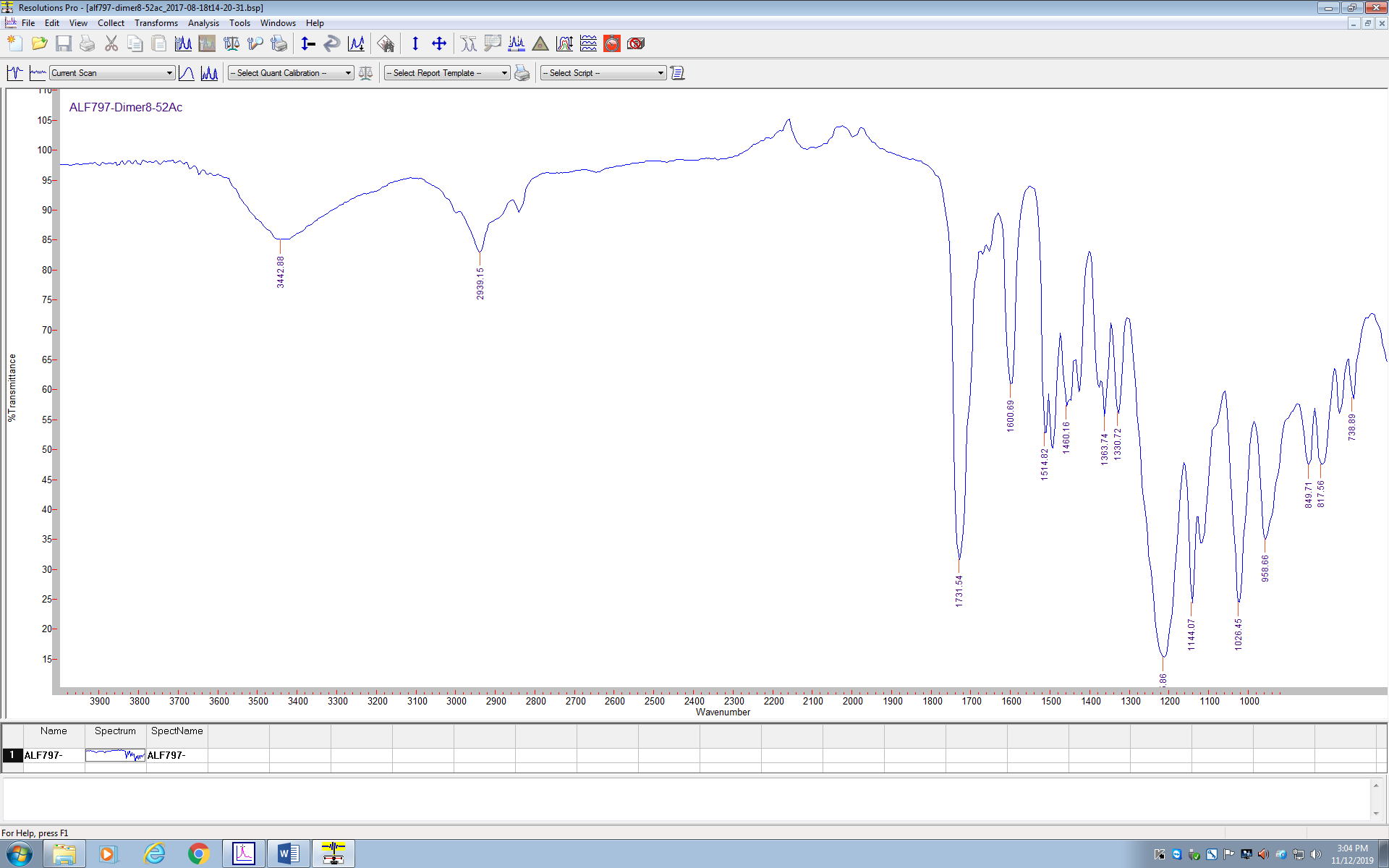


# ^1^H, ^13^C, Cosy, HSQC, HMBC NMR and FT-IR spectra Compound 9

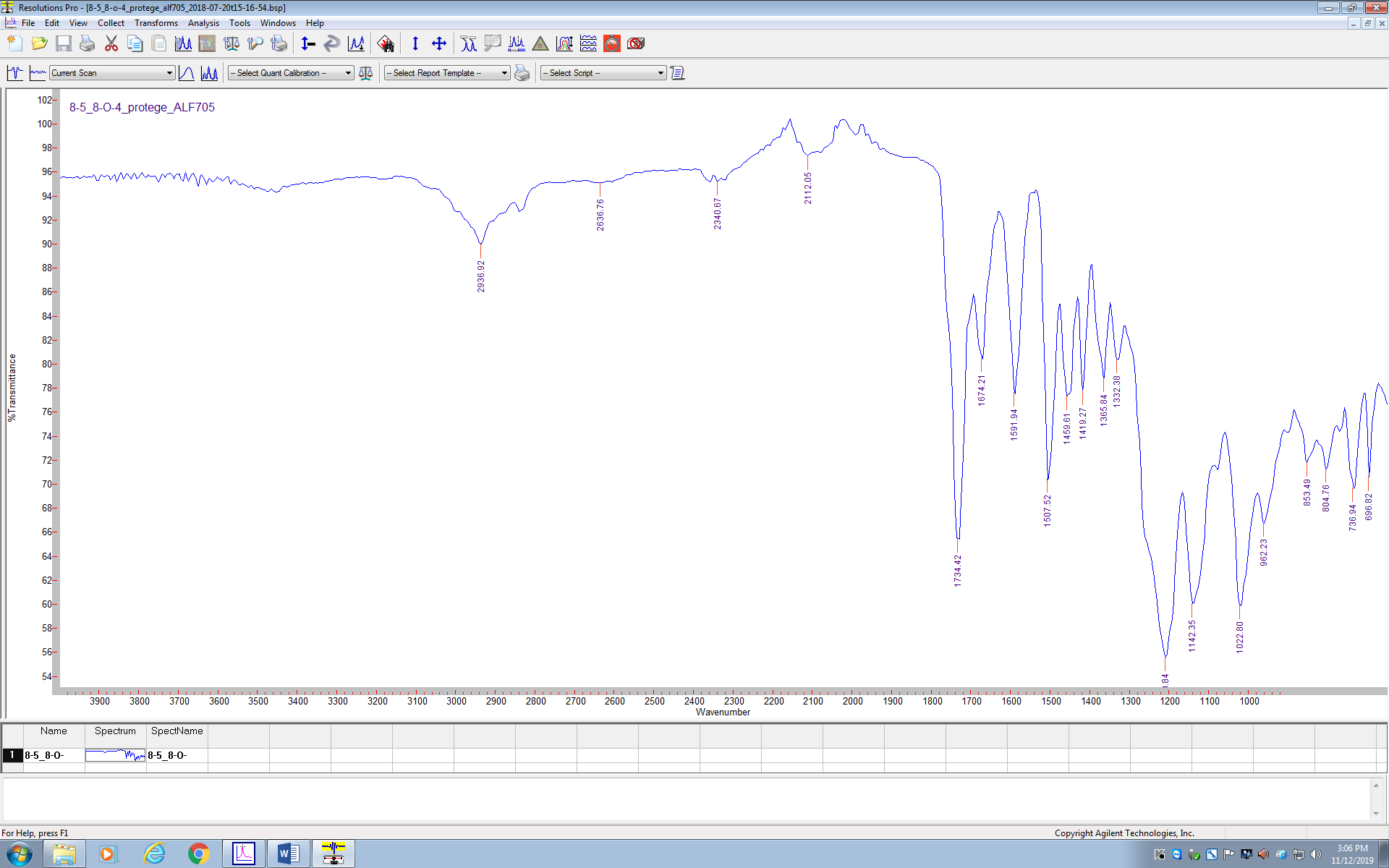


# ^1^H, ^13^C, Cosy, HSQC, HMBC NMR and FT-IR spectra Compound 10

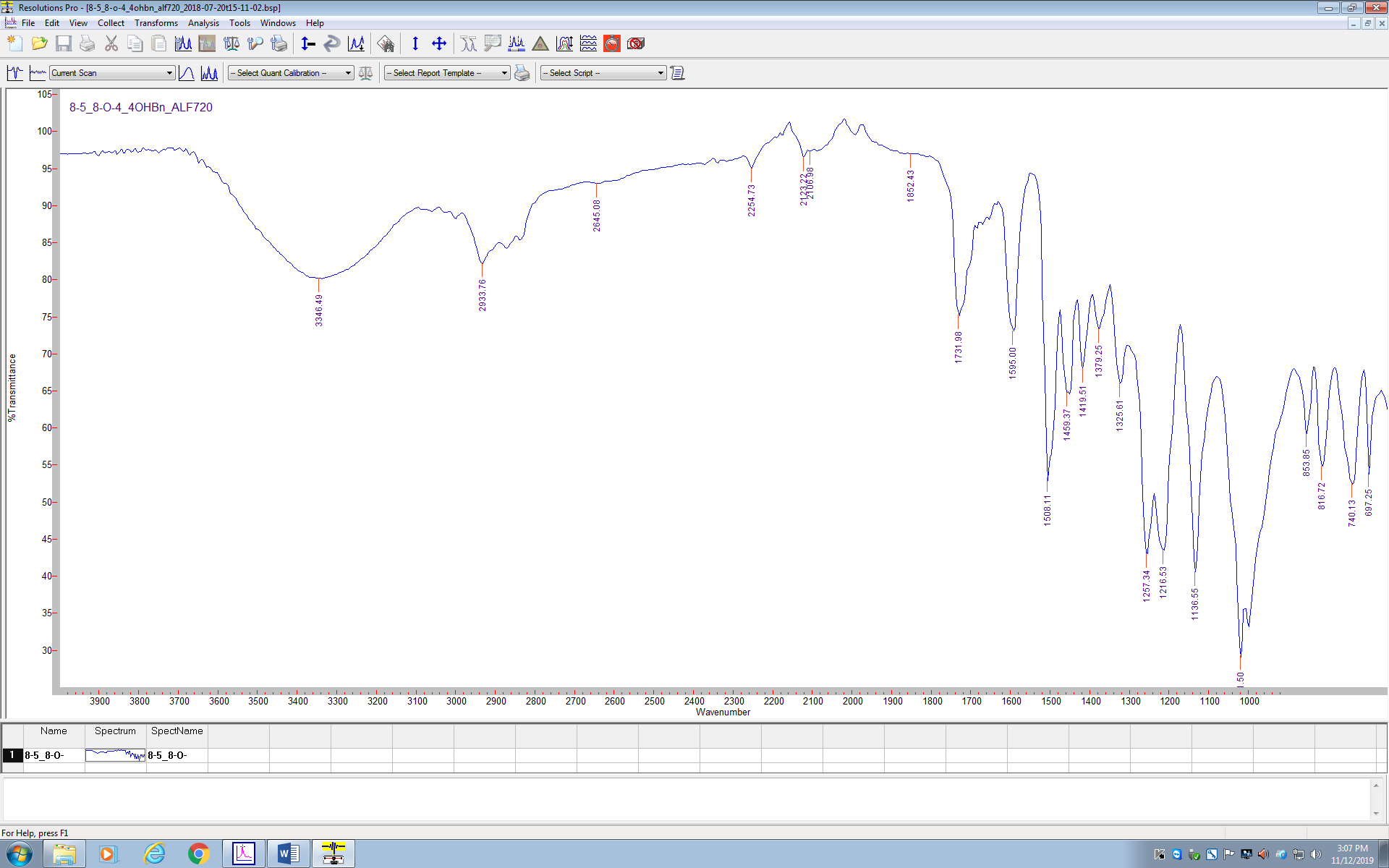


# ^1^H, ^13^C, Cosy, HSQC, HMBC NMR and FT-IR spectra Compound 11

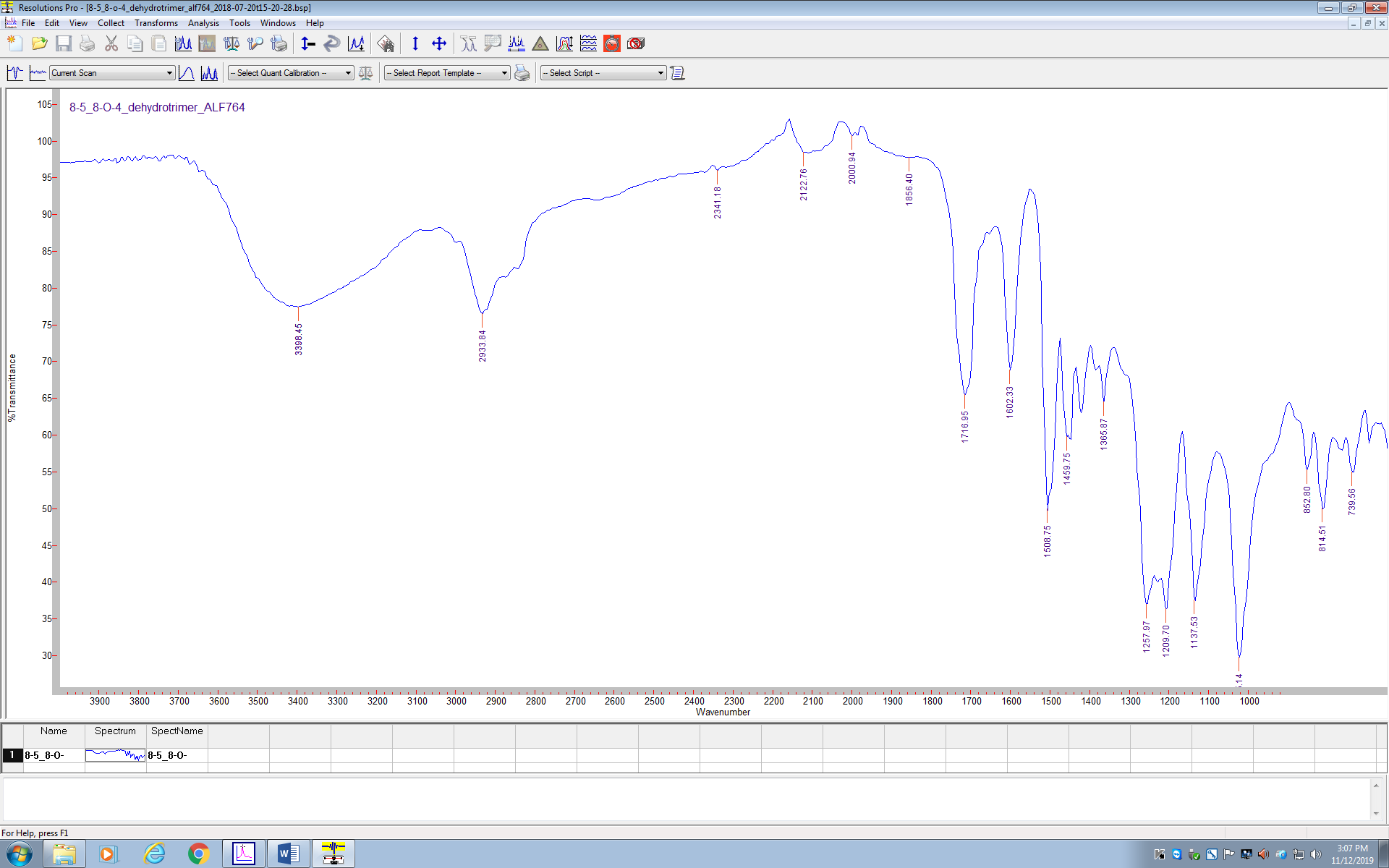


# ^1^H, ^13^C, Cosy, HSQC, HMBC NMR and FT-IR spectra Compound 15

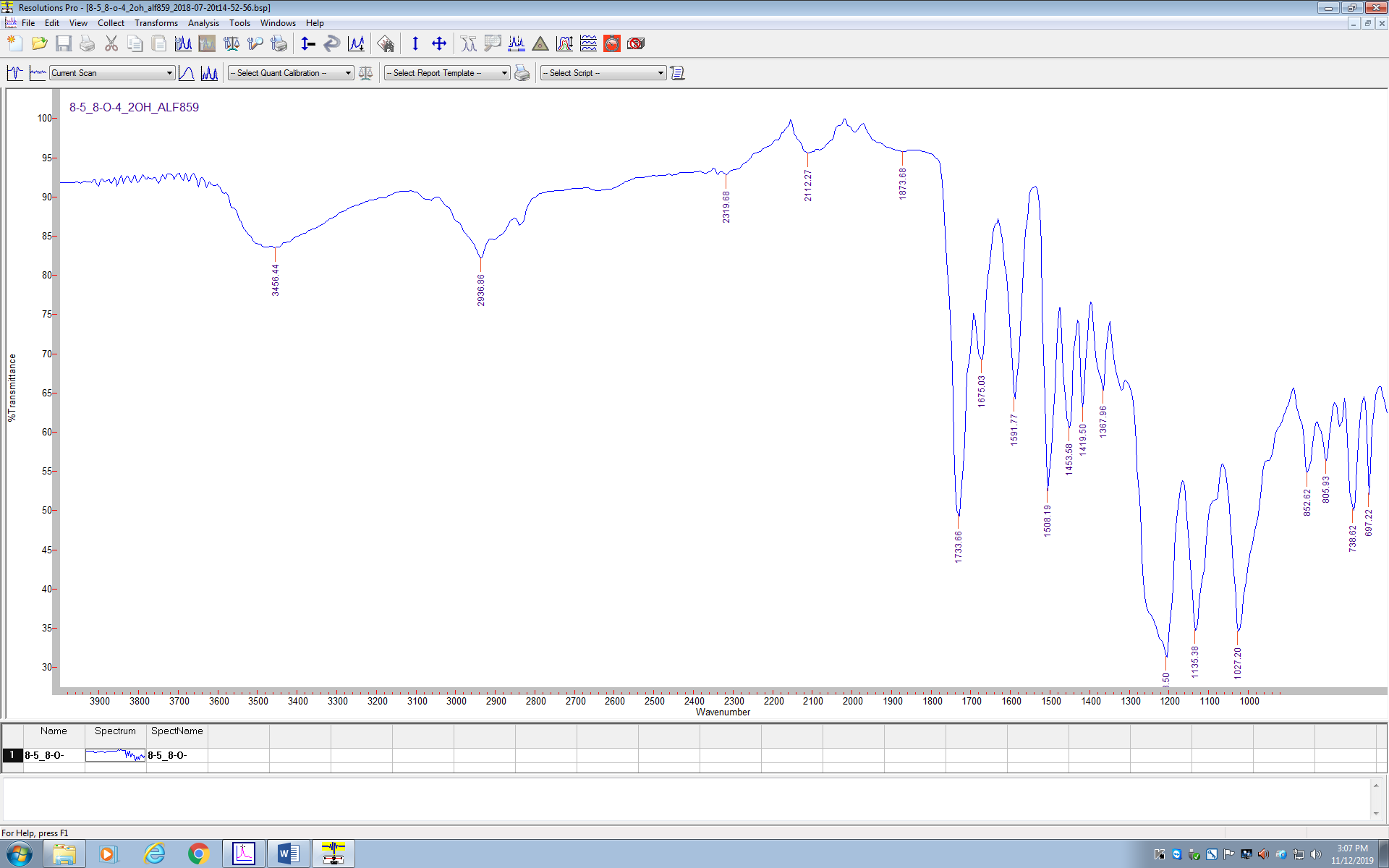


# ^1^H, ^13^C, Cosy, HSQC, HMBC NMR and FT-IR spectra Compound 16

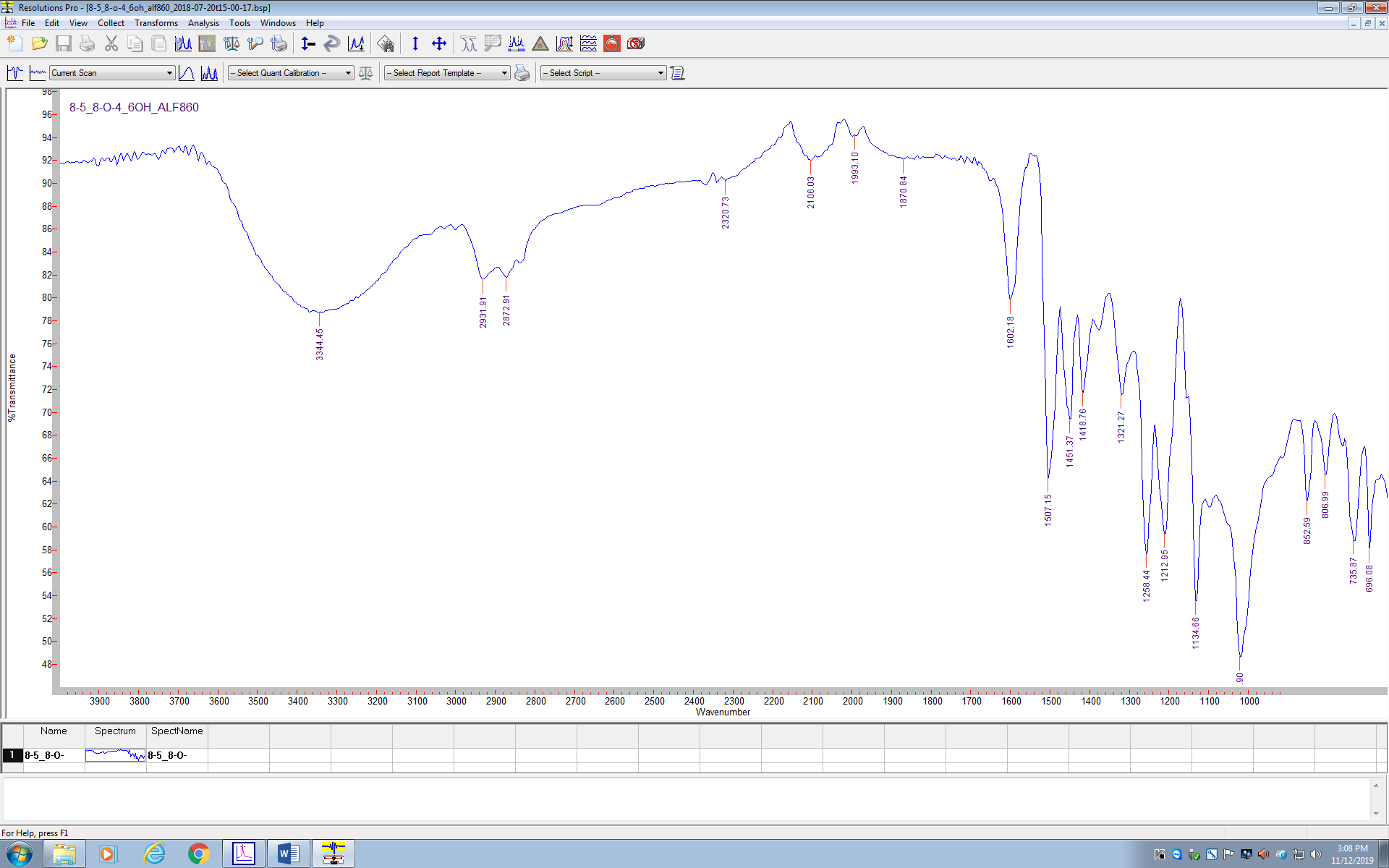


# ^1^H, ^13^C, Cosy, HSQC, HMBC NMR and FT-IR spectra Compound 14

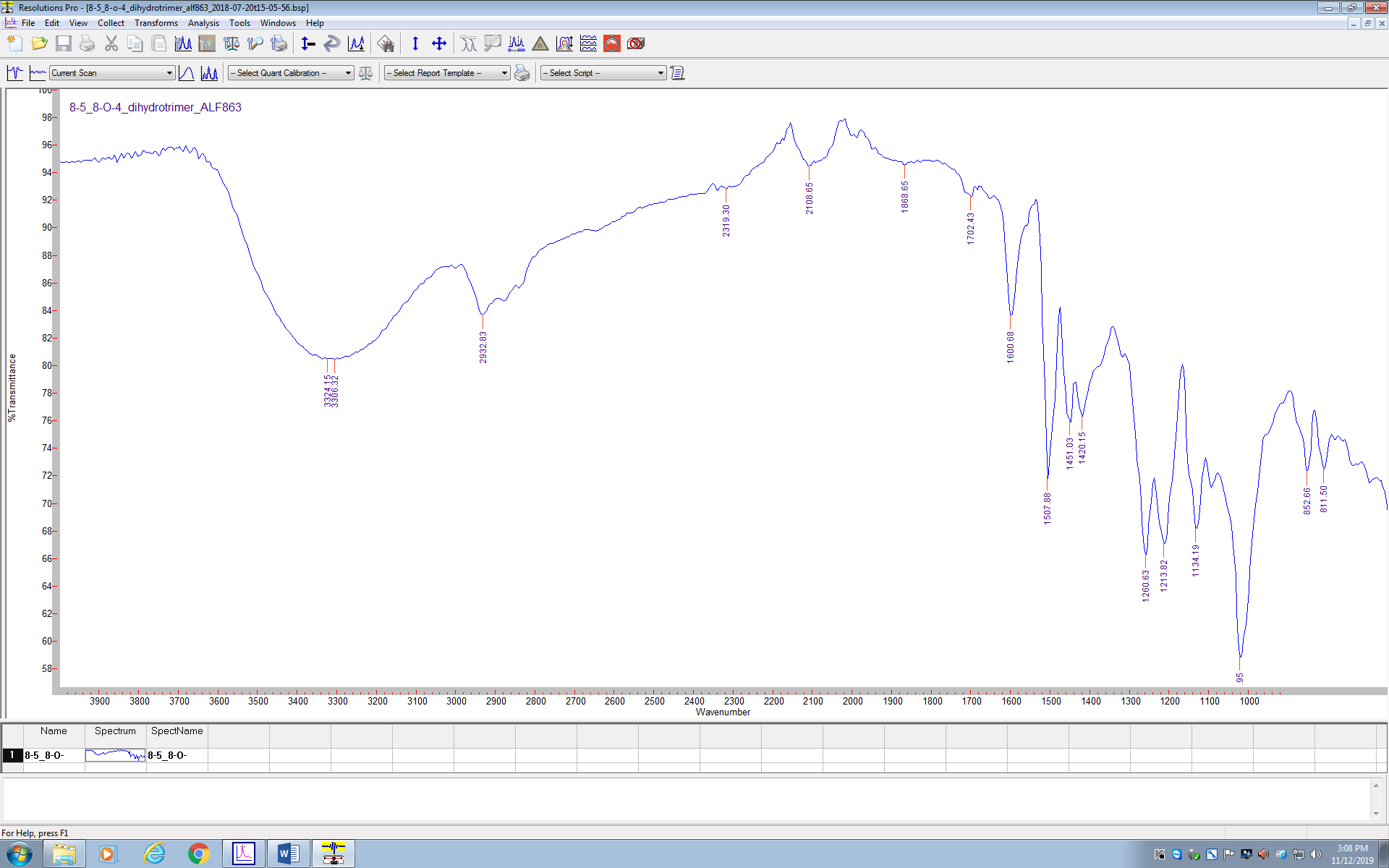


**
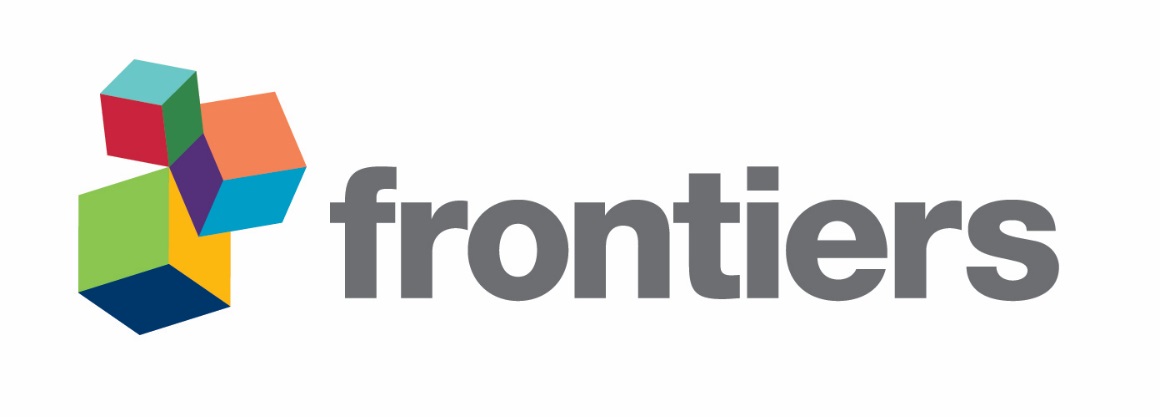
**
